# Impact of Climate Change on Distribution of Endemic Plant Section *Tuberculata* (*Camellia* L.) in China: MaxEnt Model-Based Projection

**DOI:** 10.3390/plants13223175

**Published:** 2024-11-12

**Authors:** Xu Xiao, Zhi Li, Zhaohui Ran, Chao Yan, Juyan Chen

**Affiliations:** 1 College of Forestry, Guizhou University, Guiyang 550025, China; xiaoxu199801@163.com (X.X.); ranzhaohui1998@outlook.com (Z.R.); godblessuuus@126.com (C.Y.); 2 Key Laboratory of National Forestry and Grassland Administration on Biodiversity Conservation in Karst Mountainous Areas of Southwestern China, Guizhou Academy of Forestry, Guiyang 550005, China; jychen2008@163.com

**Keywords:** MaxEnt model, sect. *Tuberculata*, potential habitat area, climate change

## Abstract

Sect. *Tuberculata*, as one of the endemic plant groups in China, belongs to the genus *Camellia* of the Theaceae family and possesses significant economic and ecological value. Nevertheless, the characteristics of habitat distribution and the major eco-environmental variables affecting its suitability are poorly understood. In this study, using 65 occurrence records, along with 60 environmental factors, historical, present and future suitable habitats were estimated using MaxEnt modeling, and the important environmental variables affecting the geographical distribution of sect. *Tuberculata* were analyzed. The results indicate that the size of the its potential habitat area in the current climate was 1.05 × 10^5^ km^2^, and the highly suitable habitats were located in Guizhou, central-southern Sichuan, the Wuling Mountains in Chongqing, the Panjiang Basin, and southwestern Hunan. The highest probability of presence for it occurs at mean diurnal range (bio2) ≤ 7.83 °C, basic saturation (s_bs) ≤ 53.36%, temperature annual range (bio7) ≤ 27.49 °C, −7.75 °C < mean temperature of driest quarter (bio9) < 7.75 °C, annual UV-B seasonality (uvb2) ≤ 1.31 × 10^5^ W/m^2^, and mean UV-B of highest month (uvb3) ≤ 5089.61 W/m^2^. In particular, bio2 is its most important environmental factor. During the historical period, the potential habitat area for sect. *Tuberculata* was severely fragmented; in contrast, the current period has a more concentrated habitat area. In the three future periods, the potential habitat area will change by varying degrees, depending on the aggressiveness of emissions reductions, and the increase in the potential habitat area was the largest in the SSP2.6 (Low-concentration greenhouse gas emissions) scenario. Although the SSP8.5 (High-concentration greenhouse gas emissions) scenario indicated an expansion in its habitat in the short term, its growth and development would be adversely affected in the long term. In the centroid analysis, the centroid of its potential habitat will shift from lower to higher latitudes in the northwest direction. The findings of our study will aid efforts to uncover its originsand geographic differentiation, conservation of unique germplasms, and forestry development and utilization.

## 1. Introduction

Global climate change is the greatest challenge facing mankind and the earth’s ecosystems in the 21st century [1]. As climate strongly influences plant growth and proliferation, it is the determining factor for the geographic range of plant species [2]. Some studies have found that the ranges of species have shifted toward higher altitudes or latitudes in response to climate change [3,4]. However, changes in the climate niches of species are usually outpaced by climate change. Consequently, climate change has caused habitat loss and created geographic barriers to the dispersal of species. This is especially pertinent to species with narrow habitat ranges, which have been forced to change their geographic range to adapt to new climatic regimes [5].

Species distribution models (SDMs) are important tools for ecological and biogeographic research and are typically used to match a species to their ecological niche factors [6]. SDMs also play an important role in the prediction of potential ranges of species based on the changes in environmental factors. The maximum entropy (MaxEnt) model is intuitive, accurate, and relatively easy to use. Over the past few decades, numerous studies have been conducted on the prediction of species distributions, detection of habitat fragmentation, novel ecological modeling methodologies, biodiversity prediction, and the effects of climate change on terrestrial biodiversity [7,8].

Sect. *Tuberculata* H.T. Chang belongs to the genus *Camellia* L. and is named after its “ovary and pericarp, both of which are markedly tuberculate”. *Callima tuberculata* Chien was discovered by the famous botanist Prof. Qian Chongping in Sichuan, China and first reported in 1939 [9]. Subsequently, in 1981, Chang Hongda first proposed the group of *Callima tuberculata* Chien based on its original traits, and categorized it into the six species discovered. To date, 18 species have been reported from this group [10,11]. Sect. *Tuberculata* is endemic to the southwest and parts of southern China, mainly in the karst landscape of southwest China, with Guizhou as the center of distribution and spreading to neighboring provinces. It grows mostly in limestone zones, showing strong regionalization, and is one of the representative taxa of typical karst regions [12,13,14]. Therefore, the evolution, distribution, and dispersal of sect. *Tuberculata* have been of interest to researchers, including phylogeny, community characteristics, and other aspects [15,16,17,18,19]. However, little has been reported on the potential distribution areas of sect. *Tuberculata* under climate change, with some reports focusing more on resource surveys and zonation studies of some of the important taxa of this group of plants at provincial scales, within nature reserves, or at smaller regional scales such as mountain systems [20,21]. As an endemic taxon of the genus *Camellia* that maintains its original traits, sect. *Tuberculata* has gone through a long evolutionary history. Predicting the spatial change pattern of its potential release area based on the species distribution model and analyzing the important environmental factors affecting its geographic distribution are of great scientific significance for the protection and conservation of reconstructing the endemic vegetation in this region.

The geographical distribution ranges of the species and their trends in different periods under different climatic conditions show similarities and differences due to environmental changes. The prediction model of a suitable area can reveal the future trend of sect. *Tuberculata* and its main environmental influence factors. Therefore, in this study, 18 sect. *Tuberculata* plants that have been reported were selected, and, based on the optimized MaxEnt model and ArcGIS v10.8 software, sect. *Tuberculata* plants were simulated from the Last interglacial (LIG), Last glacial maximum (LGM), Mid-Holocene (MH), Contemporary (Current), 2020–2040 (2030), 2040–2060 (2050), 2060–2080 (2070), and 2080–2100 (2090) geographic distributions and spatial change patterns. This will reveal the environmental impact factors affecting the distribution of sect. *Tuberculata* plants and their possible habitat areas, with a view to providing a theoretical basis for the in-depth field investigation, conservation, development, and utilization of sect. *Tuberculata*, and, at the same time, providing an auxiliary basis of geographical distribution for the study of this group of plants in the phylogenetic classification.

## 2. Results

### 2.1. Model Optimization and Accuracy Validation

**Figure 1:**
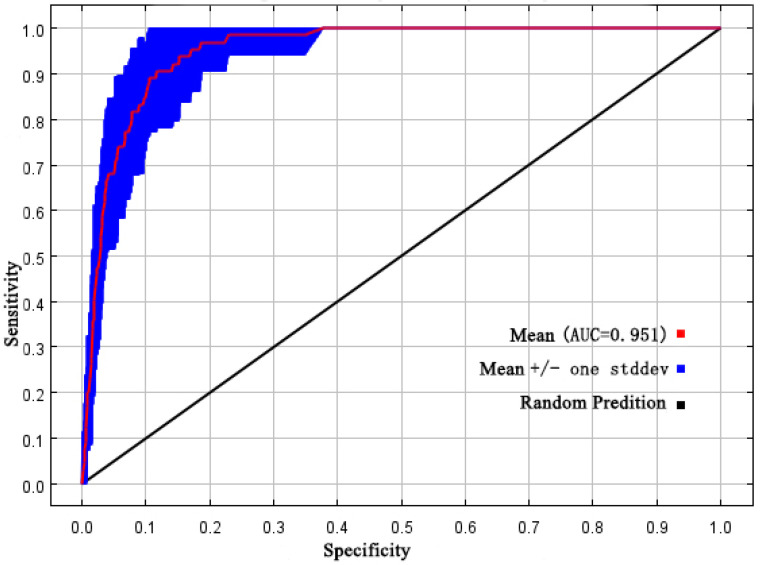
The verification of the ROC curve of prediction for sect. *Tuberculata* by the MaxEnt model.

The default settings of the MaxEnt model were generated by conducting trials to fit the 65 occurrence points of sect. *Tuberculata* plants [22]. We found that RM = 2 and FC = QP minimized the complexity and overfitting and maximized the accuracy. The accuracy of the model was then evaluated through its ROC curve. The average AUC was 0.951 ± 0.15 for all historical, current, and future climate scenarios (Figure 1), indicating that the predictions of the optimized MaxEnt model produce a smooth ROC curve with an AUC of ≥0.9. Hence, the fine-tuned model can produce reasonable predictions of the response of sect. *Tuberculata* to climate change (in terms of the geographic range), and accurately predict its potential distribution. This result also shows that the MaxEnt model can produce highly credible predictions of the potential range of sect. *Tuberculata* plants.

### 2.2. The Most Influential Climatic Factors for the Geographic Range of Sect. Tuberculata Plants

**Figure 2:**
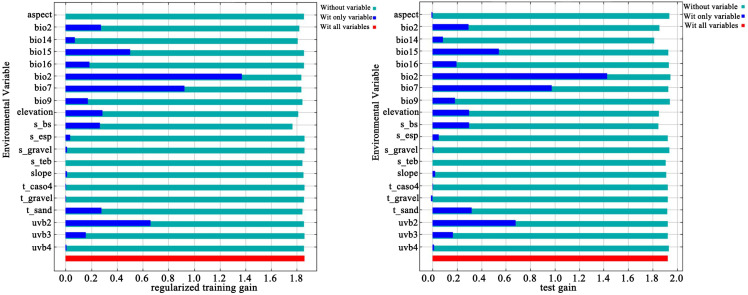
Evaluation of major environmental factors by the jackknife method.

**Figure 3:**
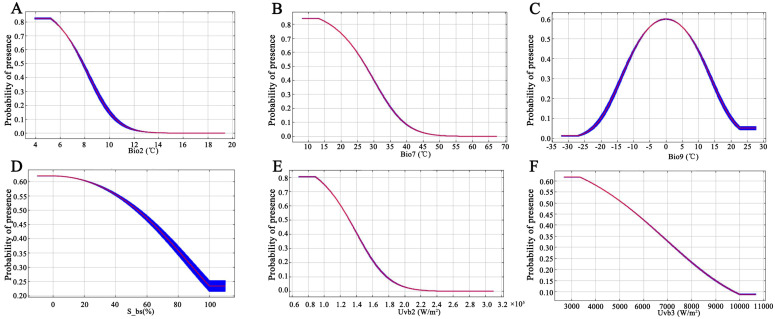
Relationship between potential suitable areas and single factor response variables. (**A**), Mean diurnal range; (**B**), Subsoil base saturation; (**C**), Annual temperature range; (**D**), Mean temperature of the driest quarter; (**E**), Seasonality of UV-B; (**F**), Mean UV-B of the highest month.

The most important climatic factors for the geographic range of sect. *Tuberculata* plants were determined from the output of the MaxEnt model, jackknife test of regularized training gain contribution, permutation importance, and univariate response curves (Table 1, Figure 2 and Figure 3). As presented in Table 1 and shown in Figure 2, the gain contributions of the mean diurnal temperature range (bio2), subsoil base saturation (S_bs), annual temperature range (bio7), mean temperature of the driest quarter (bio9), seasonality of UV-B (uvb2), and mean UV-B of the highest month (uvb3) were 46.9%, 10.4%, 10.1%, 8.9%, 7.1%, and 3.4%, respectively (86.8% cumulatively). Therefore, these six factors are the most important environmental factors for the range of sect. *Tuberculata*.

Based on the above six dominant environmental factors, single-factor simulations were carried out, and the single-factor response curves characterized the relationship between the predicted species’ pure presence probability and each variable, which can clearly reveal the correlation between the presence probability of the suitable area of the plants in sect. *Tuberculata* and the dominant factors. It is generally considered that a presence probability greater than 5% can be regarded as the most favorable range for species growth [23]. From the single-factor response curves (Figure 3), it can be seen that the probability of the existence of sect. *Tuberculata* is 0 when the average daily difference in temperature (bio2) is greater than 13.2 °C, and its existence probability increases with the decrease in bio2, and the probability of the sect. *Tuberculata* distribution reaches its peak when bio2 equals to 5.5 °C, which is the optimal fitness for submission. Taking the existence probability greater than 0.5 as the optimal fitness range, the optimal fitness range of sect. *Tuberculata* in bio2 is less than 7.83 °C. Similarly to bio2, the sect. *Tuberculata* existence probability increased with the decrease in bio7, S_bs, uvb2, and uvb3, with the optimal peaks of 13.1 °C, 0, 0.89 × 10^5^ W/m^2^, and 3350 W/m^2^, respectively, and the optimal ranges of bio7 less than 27.49 °C, S_bs less than 53.36%, uvb2 less than 1.31 × 10^5^ W/m^2^, S_bs less than 53.36%, uvb3 less than 1.31 × 10^5^ W/m^2^, and uvb3 less than 5089.61 W/m^2^. When bio9 is equal to −33 °C, the distribution probability of sect. *Tuberculata* is infinitely close to 0, and its existence probability is improved with the increase in bio9, etc. The distribution probability of sect. *Tuberculata* reaches its peak when bio9 is equal to 0 °C, which is the optimal condition for survival, and then, with the continued increase in bio9, the existence probability is reduced gradually, and it is extremely low when it reaches 22.5 °C. The existence probability is extremely low, so the optimal survival range of sect. *Tuberculata* in bio9 is −7.75 to 7.75 °C.

**Table 2:** Potential habitat areas for sect. *Tuberculata* in different periods.

Climate Scenarios		High (×10^5^ km^2^)	Medium (×10^5^ km^2^)	Low (×10^5^ km^2^)
Last Interglacial		0.01	0.12	0.5
Last Glacial Maximum		0.01	0.05	0.1
Mid Holocene		0.01	0.06	0.09
Current		0.21	0.35	0.49
2030s	SSP2.6	0.21	0.35	0.53
	SSP4.5	0.12	0.26	0.49
	SSP8.5	0.16	0.3	0.51
2050s	SSP2.6	0.18	0.38	0.58
	SSP4.5	0.19	0.43	0.58
	SSP8.5	0.22	0.41	0.6
2070s	SSP2.6	0.17	0.31	0.54
	SSP4.5	0.16	0.32	0.57
	SSP8.5	0.07	0.2	0.48
2090s	SSP2.6	0.21	0.41	0.54
	SSP4.5	0.12	0.33	0.66
	SSP8.5	0.1	0.3	0.62

### 2.3. Potential Habitat of Sect. Tuberculata Species in the Current Climate

**Figure 4:**
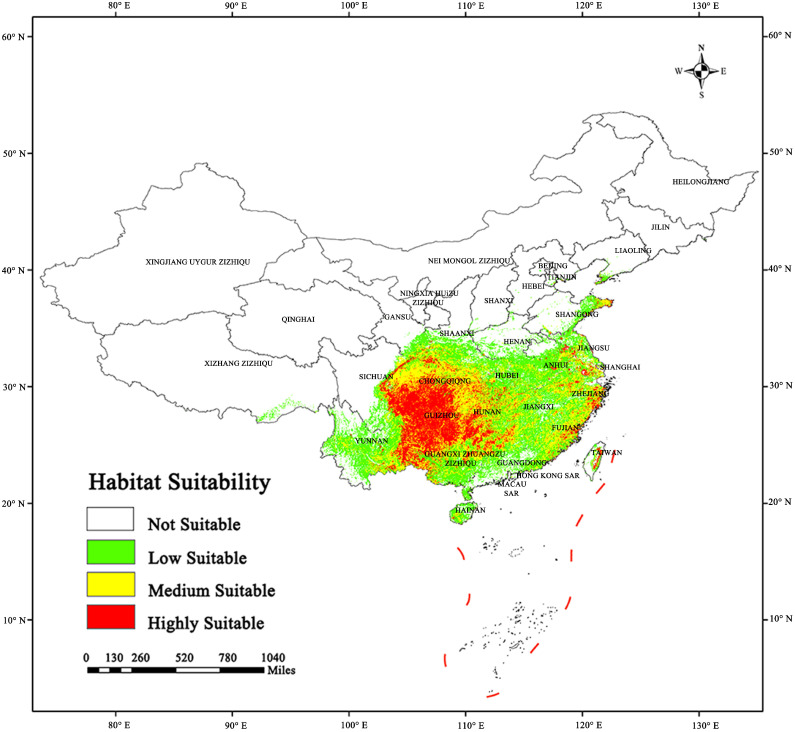
The potential habitat areas of sect. *Tuberculata* plants in China under the current climate.

The results of our study (Figure 4 and Table 2) showed that sect. *Tuberculata* plants are largely distributed between 22.54°–30.96° N and 103.83°–111.76° E. In the current climate (Figure 4), their total habitat area is 1.05 × 10^5^ km^2^, and their main area of distribution is southwestern China and some parts of China’s coastline.

High-suitability areas: these areas are mainly located in Guizhou, central and southern Sichuan, central and southern Chongqing, located at the junction of Guangxi, Yunnan and Guizhou provinces and a small part of Hunan China. The total area of high-suitability areas is 0.21 × 10^5^ km^2^, and they account for 20% of the total habitat area. Medium-suitability areas: these areas are generally located around high-suitability areas, and include eastern Yunnan, northeastern Sichuan, northern Chongqing, the border between Hunan and Hubei, central and northern Guangxi, central Fujian, and eastern-central Zhejiang. The total area of medium-suitability areas is 0.35 × 10^5^ km^2^, and they account for 33.33% of the total habitat area. Low-suitability areas: these areas are located in central Yunnan, Hainan, southern Guangxi, spots in northern Guangdong, the Qin Mountains in southern Shaanxi, the Dabie Mountains in eastern-central Hubei, eastern Hunan, the entirety of Jiangxi, central Anhui, southern Henan, and southern Xizang. The total area of low-suitability areas is 0.49 × 10^5^ km^2^, and they account for 46.67% of the total habitat area.

### 2.4. Simulation of Potential Habitat Areas for Sect. Tuberculata Plants in the Past and Future

**Figure 5:**
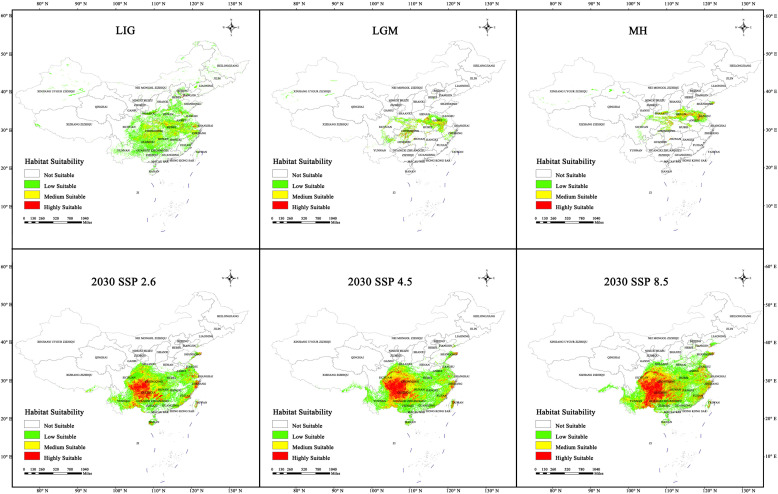
Distribution changes of the suitability of sect. *Tuberculata* in historical period and 2030. (LIG, Last Interglacial; LGM, Last Glacial Maximum; MH, Mid Holocene; SSP2.6, Low-concentration greenhouse gas emissions; SSP4.5, Medium-concentration greenhouse gas emissions; SSP8.5, High-concentration greenhouse gas emissions).

The potential distribution of sect. *Tuberculata* plants was predicted for eight different periods. Based on the results (Table 2 and Figure 5), we found that the total habitat area of sect. *Tuberculata* decreased from 0.63 × 10^5^ km^2^ in the LIG to 0.16 × 105^5^ km^2^ in the LGM, remained at 0.16 × 10^5^ km^2^ in the MH and pre-modern period, and subsequently increased to 1.05 × 10^5^ km^2^ in the current period. Therefore, the potential habitat area decreased after the LIG, stabilized during the LGM and MH, and subsequently rapidly increased in the current period. The area of highly suitable habitats did not change throughout these periods, but the areas of the medium- and low-suitability habitats gradually decreased from the LIG to the MH.

**Figure 6:**
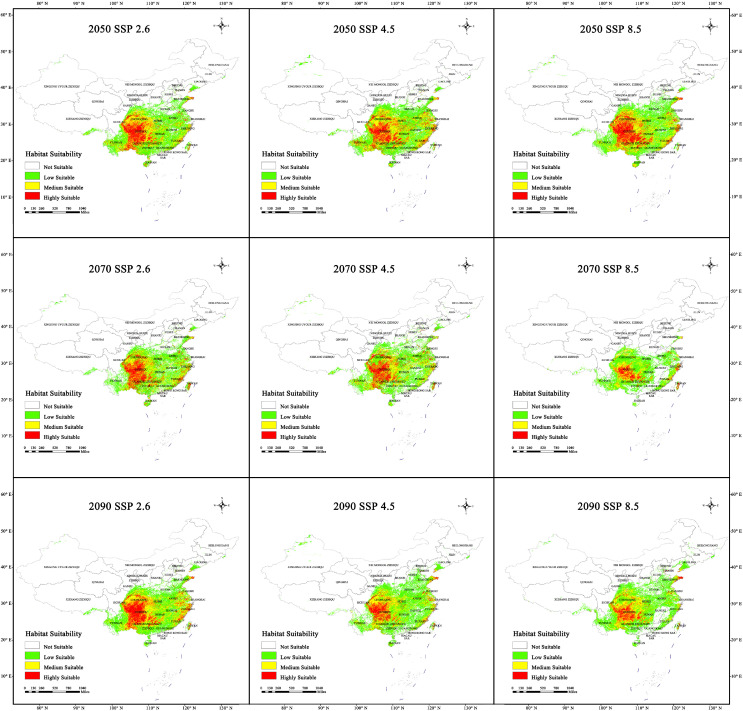
Distribution changes of the suitability of sect. *Tuberculata* in 2050, 2070, and 2090. (SSP2.6, Low-concentration greenhouse gas emissions scenario; SSP4.5, Medium-concentration greenhouse gas emissions scenario; SSP8.5, High-concentration greenhouse gas emissions).

As presented in Table 3 and shown in Figure 6, the total habitat area was predicted to decrease in the future. In all three scenarios for 2030, the total habitat area will stay greater than 0.87 × 10^5^ km^2^. In the SSP2.6 scenario, the total habitat and high-suitability areas were predicted to be 1.09 × 10^5^ and 0.21 × 10^5^ km^2^, respectively, which are similar to those in the current period. In the SSP4.5 scenario, the total habitat and high-suitability areas will be 0.87 × 10^5^ and 0.12 × 10^5^ km^2^, respectively, which are 0.18 × 10^5^ and 0.09 × 10^5^ km^2^ less than those in the current period. In the SSP8.5 scenario, the total habitat and high-suitability areas will decrease by 0.05 × 10^5^ and 0.08 × 10^5^ km^2^ compared with those in the current period to 0.97 × 10^5^ and 0.16 × 10^5^ km^2^, respectively.

In all three scenarios for 2050 (Figure 6), the total habitat area was predicted to remain above 1.14 × 10^5^ km^2^. In the SSP2.6 scenario, the total habitat area will be 1.14 × 10^5^ km^2^, which is 0.09 × 10^5^ km^2^ larger than that of the current period. The high-suitability area will be 0.18 × 10^5^ km^2^, which is 0.03 × 10^5^ km^2^ less than that of the current period. In the SSP4.5 scenario, the total habitat area will be 1.2 × 10^5^ km^2^ (0.15 × 10^5^ km^2^ higher than that of the current period), whereas the high-suitability area will be 0.19 × 10^5^ km^2^ (0.02 × 10^5^ km^2^ less than that of the current period). In the SSP8.5 scenario, the total habitat and high-suitability areas will be 1.23 × 10^5^ and 0.22 × 10^5^ km^2^, namely, 0.18 × 10^5^ and 0.01 × 10^5^ km^2^ higher than those of the current period, respectively.

In all three scenarios for 2070 (Figure 6), the total habitat area will remain above 0.75 × 10^5^ km^2^. In the SSP2.6 scenario, the total habitat and high-suitability areas will be 1.02 × 10^5^ and 0.17 × 10^5^ km^2^, which are 0.03 × 10^5^ and 0.04 × 10^5^ km^2^ less than those of the current period. In the SSP4.5 scenario, the total habitat area will be 1.05 × 10^5^ km^2^ (unchanged from that of the current period), whereas the high-suitability area will be 0.16 × 10^5^ km^2^ (0.05 × 10^5^ km^2^ less than that of the current period). In the SSP8.5 scenario, the total habitat and high-suitability areas will be 0.75 × 10^5^ and 0.07 × 10^5^ km^2^, namely, 0.3 × 10^5^ and 0.14 × 10^5^ km^2^ less than those of the current period, respectively.

In all three scenarios for 2090 (Figure 6), the total habitat area will remain above 1.02 × 10^5^ km^2^. In the SSP2.6 scenario, the total habitat area will be 1.16 × 10^5^ km^2^ (0.11 × 10^5^ km^2^ higher than that of the current period), while the high-suitability area will be 0.21 × 10^5^ km^2^ (unchanged from that of the current period). In the SSP4.5 scenario, the total habitat area will be 1.11 × 10^5^ km^2^ (0.06 × 10^5^ km^2^ higher than that of the current period), whereas the high-suitability area will be 0.12 × 10^5^ km^2^ (0.09 × 10^5^ km^2^ less than that of the current period). In the SSP8.5 scenario, the total habitat and high-suitability areas will be 1.02 × 10^5^ and 0.1 × 10^5^ km^2^, namely, 0.3 × 10^5^ and 0.11 × 10^5^ km^2^ less than those of the current period, respectively.

In summary, the potential high-suitability area for sect. *Tuberculata* will be less than those of the current period in all 12 future climate scenarios. However, the habitat areas predicted for the SSP8.5 scenarios were generally lower than those predicted for the SSP2.6 scenarios. As shown in Figure 6, the most suitable habitats for it varied from one period to another by differing degrees. Nonetheless, the geographical range was always located in the karst region in southwestern China and centered on Guizhou, from which it dispersed to other adjacent provinces. Additionally, although the potential habitat areas were relatively contiguous in the current period, they could become fragmented owing to human activity or climate change. Nonetheless, in the four future periods, habitat fragmentation appeared to be relatively mild.

### 2.5. Changes in the Spatial Pattern of Sect. Tuberculata Habitats

**Figure 7:**
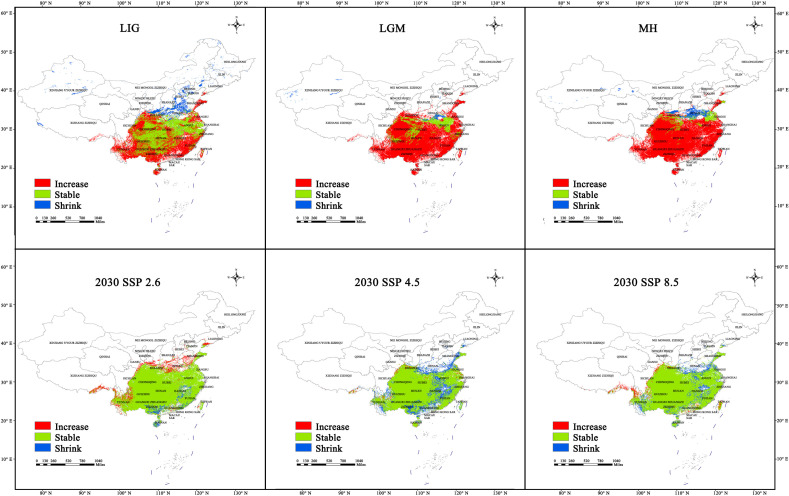
Changes in the spatial pattern of the habitats of sect. *Tuberculata* in the historical period and in 2030. (LIG, Last Interglacial; LGM, Last Glacial Maximum; MH, Mid-Holocene; SSP2.6, Low-concentration greenhouse gas emissions scenario; SSP4.5, Medium-concentration greenhouse gas emissions scenario; SSP8.5, High-concentration greenhouse gas emissions).

From the LIG to the MH, the size of the habitat of sect. *Tuberculata* initially decreased and subsequently increased (Table 3 and Figure 7). During the LIG, a habitat area of 12.0 × 10^5^ km^2^ (22.93%) was added, whereas that of 1.86 × 10^5^ km^2^ (12.45%) was lost. The newly added habitats were in central and eastern Yunnan, eastern Sichuan, southern Guangxi, northern Guangdong, Fujian, Hainan, and southern Jiangxi. The lost habitats were located in the Qinling Mountains of Shaanxi and the junction between Henan, Hebei, and Shandong. During the LGM, new habitat areas amounted to 18.92 × 10^5^ km^2^, whereas 0.37 × 10^5^ km^2^ of old habitat was lost. Therefore, the habitat of sect. *Tuberculata* underwent large changes during the cold climatic conditions of the LGM, especially in southwestern China. Predictions based on the MaxEnt model indicate that sect. *Tuberculata* has emerged in new habitats in Guangxi, Guizhou, eastern Sichuan, Chongqing, Hunan, southern Hubei, western Chongqing, and northern Jiangxi in China. Therefore, the karst region in southwest China may be a natural refuge for sect. *Tuberculata*. The lost habitat areas were mainly located in Henan and the border between Anhui and Hubei. During the MH, new, retained, and lost habitats amounted to 20.31 × 10^5^, 1.16 × 10^5^, and 1.75 × 10^5^ km^2^, respectively. The lost habitat areas were located in Henan, the Qilian Mountains in central Shaanxi, and the junction between Shandong, Henan, Jiangsu, and Anhui. The difference between the MH and LGM in terms of added habitat areas was small, at only 1.39 × 10^5^ km^2^.

**Table 3:** Changes in the range of sect. *Tuberculata* in each period.

Climate Scenarios	Increase (×10^5^ km^2^)	Stable (×10^5^ km^2^)	Shrink (×10^5^ km^2^)
Last interglacial	12	9.98	1.86
Last Glacial Maximum	18.92	3.14	0.37
Mid Holocene	20.31	1.75	1.16
2030sSSP2.6	2.12	20.92	1.14
2030sSSP4.5	0.4	18.05	4.01
2030sSSP8.5	0.78	19.78	2.28
2050sSSP2.6	2.64	21.33	0.73
2050sSSP4.5	3.44	21.65	0.41
2050sSSP8.5	4	21.71	0.35
2070sSSP2.6	1.33	20.22	1.84
2070sSSP4.5	2.15	19.78	2.28
2070sSSP8.5	0.31	15.6	6.46
2090sSSP2.6	2.47	21.84	0.23
2090sSSP4.5	3.2	20	2.06
2090sSSP8.5	2.97	18.3	3.76

**Figure 8:**
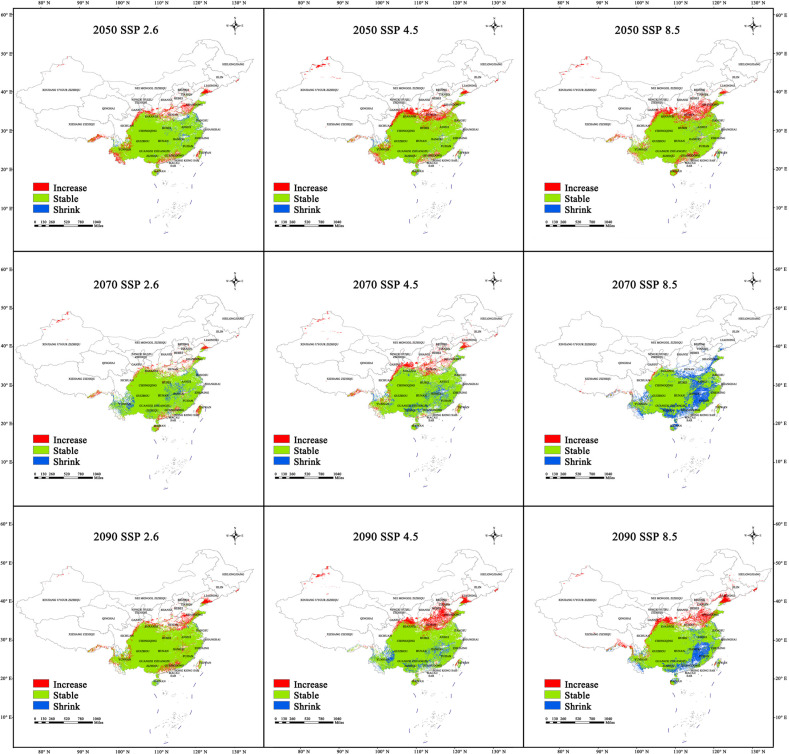
Changes in the spatial pattern of the habitats of sect. *Tuberculata* in 2050, 2070, and 2090. (SSP2.6, Low-concentration greenhouse gas emissions; SSP4.5, Medium-concentration greenhouse gas emissions; SSP8.5, High-concentration greenhouse gas emissions).

Between the 12 climate scenarios, SSP4.5 and SSP4.5 for the 2050s and 2090s, respectively, were predicted to have the largest additions in habitat area compared with the current period, at 3.44 × 10^5^ and 3.2 × 10^5^ km^2^, respectively (Figure 8). The newly added habitats will be located in the junction between Shandong, Henan, Jiangsu, and Anhui, southern Gansu, and the Qinling Mountains in central Shaanxi. Habitat addition was smallest in the SSP4.5 and SSP8.5 scenarios for the 2030s and 2070s, respectively (at 0.4 × 10^5^ and 0.31 × 10^5^ km^2^, respectively), whereas habitat loss was largest in the SSP8.5 scenario for the 2070s, at 6.46 × 10^5^ km^2^; in this scenario, the primary areas of habitat loss were predicted to be in the Wuyi Mountains (Jiangxi and Fujian), Dabie Mountains, and Qinling Mountains in eastern and northern Hubei, and parts of the Qinling Mountains in southern Shaanxi, southern Guangxi, and central Guangdong. Habitat loss was smallest in the SSP8.5 and SSP2.6 scenarios for the 2050s and 2090s, respectively (0.35 × 10^5^ and 0.23 × 10^5^ km^2^, respectively).

In summary, these predictions show that the range of sect. *Tuberculata* will become increasingly concentrated and less fragmented. The areas of habitat loss were mainly present in the Dabie Mountains and Qinling Mountains in eastern and northern Hubei, and parts of the Wuyi Mountains in Jiangxi and Fujian, which were centered on Guizhou and radiated outward to parts of the neighboring provinces. Conservation measures should be prioritized in these areas, as they are likely to be sensitive areas for habitat loss in the future.

### 2.6. Shifts in the Centroid of the Sect. Tuberculata Range

**Figure 9:**
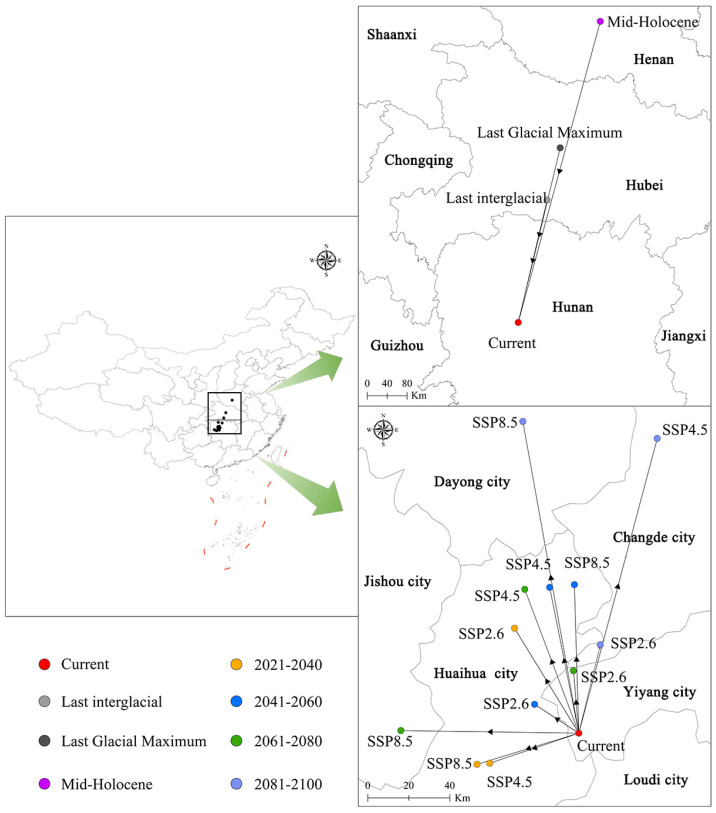
Location of the shift of the centroid of suitable areas for sect. *Tuberculata* under different climate scenarios in different periods.

Here, the shifts in the centroid of the range of sect. *Tuberculata* from one period to another were simulated by calculating its geometric center in each period [24]. As presented in Table 4 and shown in Figure 9, the centroid of the current range of sect. *Tuberculata* is located in the western part of Yiyang City in Hunan Province. During the LIG, LGM, and MH, the centroid was located 252.75, 360.18, and 623.48 km, respectively, away from its current position to the north. These shifts indirectly indicate that the climate has undergone significant changes between the LIG and MH. Overall, the centroid progressively shifted northward during these periods, but shifted southwest during the transition from the MH to the current period (Table 4 and Figure 9).

In the SSP2.6 scenario, the centroid was predicted to shift northwest in 2030, and subsequently southward from 2030 to 2050. It will then shift toward the northeast direction from 2050 to 2070 and continue to shift in this direction from 2070 to 2090. In the SSP4.5 scenario, the shifts in the centroid will be southwestward from the current period to 2030, northeastward from 2030 to 2050, westward from 2050 to 2070, and northeastward from 2070 to 2090. In the SSP8.5 scenario, the shifts in the centroid will be southwestward from the current period to 2030, northeastward from 2030 to 2050, southwestward from 2050 to 2070, and northeastward from 2070 to 2090. The largest shift in the centroid (relative to the current position) was observed in the SSP8.5 scenario for the 2090s, at 155.18 km. The shortest shift in the centroid was observed in the SSP2.6 scenario for the 2050s, at 25.40 km.

Overall, the centroid is expected to shift northwest in the future; from its current position, it will shift towards the Wuyi Mountains in northwestern Hunan. In particular, climate change will cause a shift in the range of sect. *Tuberculata* toward higher latitudes.

## 3. Discussion

### 3.1. The Most Important Environmental Factors for the Distribution of Sect. Tuberculata

The MaxEnt model is an important research tool for biodiversity, conservation, and evolution studies [25,26,27,28]. In the study, the MaxEnt model was used to predict the potential ranges of sect. *Tuberculata* plants in the past, present, and future. The six dominant environmental factors for sect. *Tuberculata* were obtained using the jackknife method, contribution rates, and univariate response curves, and were determined as bio2, S_bs, bio7, bio9, uvb2, and uvb3. The most important factor is bio2, namely, the mean diurnal temperature range. Therefore, temperature is the most important environmental factor for the range of sect. *Tuberculata*, followed by the intensity of UV radiation. In particular, the climate restricts the geographic range of these plants on the regional scale, and the most important climatic factors are hydrothermal. In a previous study on the climatic factors affecting *C. rubimuricata* in the Guizhou Maolan National Nature Reserve [29], the temperature was found to be the most important factor of all, which is consistent with our findings. Guo et al. studied the germination and physiological characteristics of *C. rubituberculata* in karst regions and found that temperature was the most important factor for seed germination, followed by illumination [30]. Hence, temperature factors are more important than illumination factors for it. Its range was mainly located in southwestern China and centered on Guizhou, from which it dispersed to adjacent provinces. Southwestern China has a wet subtropical and mountainous climate and is a classic mountain-and-valley habitat for East Asian flora, with a complex terrain typical of karst regions. Owing to this unique geology and climate, the flora of this region are usually xerophytes and lithophytes [31,32]. To achieve respiration and photosynthesis, a plant must be provided with a suitable range of temperatures, and the optimal range varies from one species to another [33]. Excessively low temperatures can lead to frost damage, which, in turn, inhibits growth [34]. Sect. *Tuberculata* is a typical karst flora whose distribution can span a wide range of environmental conditions. In phytomorphological studies, its leaves have characteristics closely related to cold and drought resistance, such as low stomatal density and trichomes on the leaves. These features allow it to have high photosynthetic and heat dissipation efficiencies, which helps them survive in harsh environments [18]. The next most important factor is UV intensity, which indirectly affects photosynthesis, thus influencing the plant’s distribution [35].

### 3.2. Changes in the Potential Habitable Range of Sect. Tuberculata Plants and Centroid Analysis

Studies regarding the potential distribution pattern of sect. *Tuberculata* plants in the presence of climate change play an important role in informing species conservation and ecosystem preservation efforts. The distribution of each species reacts differently to climate change. In this study, we simulated the possible geographic ranges of sect. *Tuberculata* at different periods using the MaxEnt model and the ArcGIS system. This is the first study of its distribution; we found that it is mainly distributed between 22.54°–30.96° N latitude and 103.83°–111.76° E longitude. This range is primarily located in southwestern China, which is consistent with previous findings. This also shows that the optimized MaxEnt model can accurately and effectively predict its range [36]. Climate plays an important role in determining the distribution of a species; hence, the distribution of some species is a reflection of the climate [5]. Based on the results, we found that sect. *Tuberculata* is mainly located in the karst highlands of Guizhou, which is consistent with the results of previous studies [14]. Between the LIG and current period, its habitat initially decreased and subsequently increased to its maximum in the current period. Therefore, its habitat has expanded to some extent during the current period. The unique climate of the karst region creates a humid and shady environment, which is an ideal habitat for sect. *Tuberculata*. Although its potential habitats were relatively contiguous during the LIG, LGM, and MH periods, they have become fragmented during the current period. We speculate that this could be caused by increased CO_2_ emissions and human activities, such as logging. In the four future periods, the habitable area for sect. *Tuberculata* will decrease by varying degrees (depending on the emissions mitigation scenario) compared with that in the current period. Therefore, it may not adapt well to the climatic conditions of the future. In the predictions, we found that the loss of habitats in the 2070s and 2090s was relatively low in the SSP2.6 scenario but large in the SSP8.5 scenario. We speculate that this is because the high-emissions SSP8.5 scenario leads to worsened environmental conditions and higher temperatures, which exacerbates fragmentation in the habitat of sect. *Tuberculata*. Therefore, we show that global warming poses a tremendous challenge to it.

According to centroid analysis, the current centroid of the sect. *Tuberculata* habitat was located in the western part of Yiyang city in Hunan. The centroid shifted northward during the LIG-MH, and subsequently shifted southwestward during the current period. Global warming was found to shift the centroid of its potential habitat toward higher latitudes, which is consistent with previous findings [37,38]. In the past, sect. *Tuberculata* gradually adapted to the wet and humid mountainous environment owing to changes in the terrain; in the future, it may evolve to migrate towards lower altitudes. However, the potential range of a plant is not only affected by climate and terrain, but also by human activities and other abiotic factors, which can also its their growth and development [39]. Therefore, future studies on the range of sect. *Tuberculata* should simultaneously consider the plant’s intrinsic physiology and ecosystem, and human factors, in their models to improve the predictive accuracy.

## 4. Materials and Methods

### 4.1. Data Collection and Processing 

**Figure 10:**
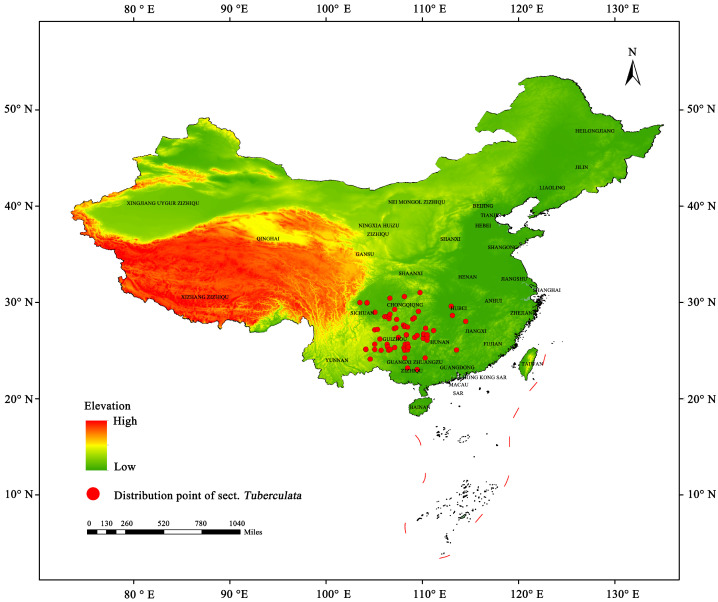
Screened occurrence points of the sect. *Tuberculata* plants.

The occurrence records of sect. *Tuberculata* plants were obtained from online platforms, such as the Chinese Virtual Herbarium (https://www.cvh.Ac.cn/, accessed on 10 February 2024), Global Biodiversity Information Facility (https://www.gbif.org/, accessed on 10 February 2024), and Plants of the World Online (https://powo.science.kew.org/,accessed on 10 February 2024). Moreover, 86 occurrence records were obtained from field surveys in 23 locations. The occurrence records were sorted by species name, longitude (X), and latitude (Y), and stored as .csv files. By keeping only one point out of all points within a range of 10 km, 71 data points were obtained. To avoid the occurrence points close to each other, the “trim duplicate occurrences” tool in ENMTools was used to exclude redundant data points in each 10 × 10 km grid. Finally, 65 valid occurrence points were obtained (Figure 10), which were stored in a .csv file and subsequently used in the MaxEnt model [40].

### 4.2. Acquisition and Selection of Environmental Data

To study the potential habitat range of sect. *Tuberculata* plants, range prediction was conducted using 60 environmental factors in eight periods, namely, the LIG, LGM, MH, current period, 2020–2040 (2030), 2040–2060 (2050), 2060–2080 (2070), and 2080–2100 (2090). The environmental factors include 19 bio-climatic factors, three terrain factors, 32 soil factors, and six ultraviolet (UV) factors. The 19 climatic factors were obtained from Worldclim (http://www.worldclim.org/, accessed on 25 February 2024) [41], which includes data for the current period (1970–2000) and projections for future periods (2021–2100). The SSP2.6, SSP4.5, and SSP8.5 (aggressive, moderate, and weak emission mitigation) climate scenarios from Community Climate System Model version 4 (CCSM4) were used as future climate projections. Version 2 of the Beijing Climate Center Climate System Model (BCC-CSM2-MR_2.5) was used as the atmospheric circulation model, as it performs well in the prediction of air temperatures and precipitation in China [42,43,44]. The terrain data were obtained from Worldclim, and the slope and aspect data were obtained through ArcGIS. The soil factors were obtained from the China dataset of the Harmonized World Soil Database (v1.1) (http://vdb3.soil.csdb.cn, accessed on 25 February 2024). The UV data were obtained from the glUV dataset (http://www.ufz.de/gluv/, accessed on 25 February 2024) [19]. In order to prevent overfitting due to multicollinearity between environmental factors, we performed a Spearman correlation test the environmental data based on the model contribution in R (4.1.1) [45]. All environmental factors with correlations higher than 0.8 and low model contributions were excluded; thus, the remaining 20 statistically and biologically significant environmental factors were used for range modeling (Table 5).

### 4.3. Building and Optimizing the Model

Optimization of MaxEnt model parameters was carried out using “kuenm” in R v3.6.3. [21]. Accordingly, parameter combinations were created by combining regularization multiplier (RM) values from 0.5 to 4 (in increments of 0.5) with six feature classes (FCs), namely, L, LQ, H, LQH, LQHP, and LQHPT (L: linear, Q: quadratic, H: hinge, P: product, T: threshold) [46,47]. The kuenm package was then used to test the parameter combinations, and the fit and complexity of each candidate model was assessed using the delta of their Akaike information criterion (AIC) values (∆AIC) [48,49]. The parameter combination resulting in the lowest ∆AIC was used to construct the MaxEnt model, and the resulting model was evaluated using the area under the curve (AUC) of its receiver operating characteristic (ROC) curve. The AUC scores were interpreted as follows: <0.6: failed, 0.6–0.7: poor, 0.7–0.8: fair, 0.8–0.9: good, and >0.9: excellent [40].

### 4.4. Model Assessment

Twenty environmental variables and 65 occurrence points were included in the MaxEnt v3.4.4 model [48,49,50]. The model parameters were configured as follows: (1) In the model, 75% of the distribution points were used as training data, while the remaining 25% were used as test data, with other settings kept at their default values. (2) The environmental response curve and predicted range map were plotted. (3) The jackknife method was used to evaluate the importance and contribution of each environmental factor, and the logistic output format was used. All other parameters remained in their default settings. Accuracy validation was conducted by evaluating the ROC AUC value (0 to 1) derived from the MaxEnt model’s output; the larger the AUC, the higher the credibility of the model’s predictions [21]. The AUC values were interpreted as follows: <0.7: poor, 0.7–0.8: fair, 0.8–0.9: good, and >0.9: excellent [42].

### 4.5. Classification of Habitat Areas

Based on the classification criterion used for sect. *Tuberculata* plants in China [50] and the output of the MaxEnt model, the potential habitats for sect. *Tuberculata* plants were classified into four types using the natural breaks method. In particular, the potential area of distribution was segmented into four areas based on the average logistic values and actual area of distribution of the plants, namely, non-suitable (0–0.2), low suitability (0.2–0.4), medium suitability (0.4–0.6), and high suitability (0.6–1) areas. Breaks were then input manually, and the total habitat area was obtained by combining the medium- and high-suitability areas.

### 4.6. Centroid Analysis

The direction and distance of species habitat shifts can be inferred from the changes in the habitat’s centroid [49]. Here, the kuenm R package was used to compute changes in the spatial pattern of the total habitat area in each period, as well as the habitat’s centroid [51]. Finally, the SDM was used to track the coordinates of the centroid, thus determining the centroid of sect. *Tuberculata* plants in each period. The calculated coordinates of the centroid were then used to determine the distance of habitat shift [52].

## 5. Conclusions

In the study, a MaxEnt model optimized by the kuenm R package was used to predict the distribution and potential habitat areas of sect. *Tuberculata* plants in three historical periods, the present, and 12 future climate change scenarios. The size of its potential habitat area in the current climate was 1.05 × 10^5^ km^2^, and the highly suitable habitats were located in Guizhou, central-southern Sichuan, the Wuling Mountains in Chongqing, the Panjiang Basin, and Southwestern Hunan. In the centroid analysis, the centroid of its potential habitat will shift from lower to higher latitudes in the northwest direction. We expect that our findings will help inform future field surveys of sect. *Tuberculata* plants, as well as their conservation and rehabilitation. Additionally, our results will act as auxiliary evidence for its geographic range in phylogenetic studies.

## Figures and Tables

**Table 1:** Importance of each dominant environment variable in the MaxEnt model.

Environmental Variables	Description	Contribution (%)	Suitable Range
Bio2	Mean diurnal range	46.9	<7.83 °C
S_bs	Subsoil base saturation	10.4	<53.36%
Bio7	Annual temperature range	10.1	<27.49 °C
Bio9	Mean temperature of the driest quarter	8.9	−7.75–7.75 °C
Uvb2	Seasonality of UV-B	7.1	<1.31 × 10^5^ W/m^2^
Uvb3	Mean UV-B of the highest month	3.4	<5089.61 W/m^2^

**Table 4:** Shifts in the centroid of the range of sect. *Tuberculata* in each period.

Index		Longitude (°)	Latitude (°)	Distance (km)
LIG		111.68	30.28	252.75
LGM		112.05	31.20	360.18
MH		113.17	33.43	623.48
Current		110.89	28.12	0.00
2030s	SSP2.6	110.61	28.60	60.06
	SSP4.5	110.44	28.02	44.76
	SSP8.5	110.38	28.02	50.58
2050s	SSP2.6	110.68	28.26	25.40
	SSP4.5	110.80	28.77	73.02
	SSP8.5	110.92	28.77	72.98
2070s	SSP2.6	110.88	28.39	30.79
	SSP4.5	110.67	28.76	75.29
	SSP8.5	110.01	28.19	84.53
2090s	SSP2.6	111.02	28.50	44.62
	SSP4.5	111.39	29.38	149.10
	SSP8.5	110.72	29.50	155.18

**Table 5:** Relative importance of each environmental factor in the MaxEnt model.

Variable	Description	Percent Contribution (%)	Permutation Importance (%)
bio2	Mean diurnal range (Mean of monthly (max temp–min temp))	46.9	14.2
s_bs	Basic saturation (lower level)	10.4	8.4
bio7	Temperature annual range	10.1	30.3
bio9	Mean temperature of driest quarter	8.9	5.4
uvb2	Annual UV-B seasonality (standard deviation)	7.1	0.6
uvb3	Mean UV-B of highest month	3.4	0
bio15	Precipitation seasonality	2.7	0.7
elevation	Altitude	2.3	16.3
t_sand	Sand content (upper layer)	2.3	1.4
bio12	Annual precipitation	2.3	10
slope	Slope	1.6	1.1
bio14	Precipitation of driest month	1.1	8
s_teb	Exchangeable salt base (lower layer)	0.3	1.3
t_gravel	Percent of gravel (upper layer)	0.3	0.3
aspect	Aspect	0.2	0.3
bio16	Precipitation of wettest quarter	0.1	1.1
uvb4	Mean UV-B of lowest month	0.1	0.4
s_gravel	Percent of gravel volume (lower layer)	0	0
s_esp	Exchangeable sodium salt (lower layer)	0	0.1
t_caso4	Sulfate content (upper layer)	0	0

## Data Availability

Data are contained within the article.
